# Sex-driven variability in TSPO-expressing microglia in MS patients and healthy individuals

**DOI:** 10.3389/fneur.2024.1352116

**Published:** 2024-02-20

**Authors:** Sini Laaksonen, Maija Saraste, Marjo Nylund, Rainer Hinz, Anniina Snellman, Juha Rinne, Markus Matilainen, Laura Airas

**Affiliations:** ^1^Turku PET Centre, Turku University Hospital, University of Turku, Turku, Finland; ^2^Neurocenter, Turku University Hospital, Turku, Finland; ^3^Clinical Neurosciences, University of Turku, Turku, Finland; ^4^InFLAMES Research Flagship, University of Turku, Turku, Finland; ^5^Wolfson Molecular Imaging Centre, University of Manchester, Manchester, United Kingdom

**Keywords:** multiple sclerosis, microglia, sex, positron emission tomography, TSPO (18 kDa translocator protein)

## Abstract

**Background:**

Males with multiple sclerosis (MS) have a higher risk for disability progression than females, but the reasons for this are unclear.

**Objective:**

We hypothesized that potential differences in TSPO-expressing microglia between female and male MS patients could contribute to sex differences in clinical disease progression.

**Methods:**

The study cohort consisted of 102 MS patients (mean (SD) age 45.3 (9.7) years, median (IQR) disease duration 12.1 (7.0–17.2) years, 72% females, 74% relapsing–remitting MS) and 76 age- and sex-matched healthy controls. TSPO-expressing microglia were measured using the TSPO-binding radioligand [^11^C](R)-PK11195 and brain positron emission tomography (PET). TSPO-binding was quantified as distribution volume ratio (DVR) in normal-appearing white matter (NAWM), thalamus, whole brain and cortical gray matter (cGM).

**Results:**

Male MS patients had higher DVRs compared to female patients in the whole brain [1.22 (0.04) vs. 1.20 (0.02), *p* = 0.002], NAWM [1.24 (0.06) vs. 1.21 (0.05), *p* = 0.006], thalamus [1.37 (0.08) vs. 1.32 (0.02), *p* = 0.008] and cGM [1.25 (0.04) vs. 1.23 (0.04), *p* = 0.028]. Similarly, healthy men had higher DVRs compared to healthy women except for cGM. Of the studied subgroups, secondary progressive male MS patients had the highest DVRs in all regions, while female controls had the lowest DVRs.

**Conclusion:**

We observed higher TSPO-binding in males compared to females among people with MS and in healthy individuals. This sex-driven inherent variability in TSPO-expressing microglia may predispose male MS patients to greater likelihood of disease progression.

## 1 Introduction

Multiple sclerosis (MS) is an autoimmune disease affecting the central nervous system (CNS). It is more common among women, with a female-to-male incidence ratio of 2.5 (1). MS is characterized by focal inflammatory lesions in white and gray matter, which leads to passing neurological symptoms, or relapses. Simultaneously with the focal inflammatory activity, which is driven by the adaptive peripheral immune system, a neurodegenerative process commences and leads to progressive, steadily worsening symptoms. Although this smoldering process can be already present in early MS, its clinical consequences become more evident later in the disease, with increasing patient age (2).

Microglia are the principal cells of the innate immune system within the CNS, and they function as gatekeepers under normal circumstances. They defend the brain against foreign intruders and clean debris from damaged cells and synapses to ensure optimal functioning of the nervous system. Microglia receive an activation signal upon detection of foreign antigens or damaged cells and alter their function and phenotype according to the trigger (3). The functional adjustments promote re-organization of the cells within the CNS tissue, leading to increased density of microglial cells at sites of tissue damage (3, 4). The term “microglial activation” encompasses many different microglial phenotypes, both pro- and anti-inflammatory, and with numerous different functions (5). Under pathological conditions, such as in the context of neurodegeneration, microglia acquire a pro-inflammatory phenotype, which may accelerate the neurodegenerative process. This is a common phenomenon in several neurodegenerative diseases including MS (6). Neuropathological studies have demonstrated increased microglial activation characterized by pro-inflammatory marker CD68 and decreased number of homeostatic microglia in the normal-appearing white matter (NAWM) of postmortem MS brain (7).

*In vivo*, microglia can be assessed using positron emission tomography (PET) imaging and radioligands binding to 18 kDa translocator protein (TSPO) (8–10). TSPO is a mitochondrial protein expressed by activated microglia/macrophages and reactive astrocytes and upregulated in neuroinflammation (8, 11). Although immunohistochemical studies have suggested that the upregulation of TSPO in neuroinflammation is mainly due to increased density of microglia (4, 11, 12), a recent re-analysis of three single-nucleus RNAseq studies (13–15) demonstrated increased TSPO expression in activated microglia with a pro-inflammatory phenotype (16). Regardless the fundamental reason of increased TSPO-PET signal in MS brain, clinical TSPO-PET studies have provided substantial evidence of its detrimental nature (17). Widespread, increased TSPO-binding was demonstrated in secondary progressive (SP) MS compared to relapsing–remitting (RR) MS and healthy controls (HC) (18). Increased TSPO-binding correlated well with brain atrophy measures, signs of microstructural and neuroaxonal damage, and with Expanded Disability Status Scale (EDSS) values (18–20). Moreover, increased TSPO-binding in the NAWM and in thalamus predicted later disease worsening (18–22).

Even though women are more susceptible to develop MS, men have a higher risk of long-term disability progression. Male sex predicts a shorter time to SPMS at a younger age, and male MS patients accumulate disability faster (23, 24). Furthermore, male MS patients have imaging evidence of neurodegeneration, supported by faster brain atrophy and a higher likelihood of development of chronic hypointense T1 lesions in brain MRI (25). Furthermore, male MS patients have more severe cognitive decline than that experienced by female patients (26, 27). However, mechanisms behind these sex differences in cognition, disease progression and brain MRI findings are not well understood.

Given the convincing role of microglial activation in promoting disease progression in MS, we hypothesized that sex differences in TSPO-expressing microglia could be accelerating the more rapid disease progression experienced by men with MS. This was studied using TSPO-PET imaging in a large cohort of MS patients and healthy volunteers, with the discovery that TSPO-binding was significantly higher in males compared to females in several regions of interest (ROI) in the brain, both in MS patients and in healthy individuals.

## 2 Materials and methods

### 2.1 Study participants and study procedures

Hundred and two MS patients were recruited between 2009 and 2022 from the outpatient clinic of the Neurocenter at Turku University Hospital in Turku, Finland. They were imaged at the Turku PET Centre (TPC) using TSPO-PET and MRI. The inclusion criteria for the study were a diagnosis of MS according to diagnostic criteria appropriate to the period of diagnosis and willingness to participate in a PET study. Exclusion criteria included clinical relapse and/or corticosteroid treatment within 30 days of evaluation, gadolinium contrast enhancement in MRI, active neurological or autoimmune disease other than MS, another notable comorbidity, pregnancy and intolerability to PET or MRI. [^11^C](R)-PK11195 data from healthy controls imaged in TPC (*n* = 37) between 2018 and 2020 or Wolfson Molecular Imaging Centre in Manchester (WMIC; *n* = 39) between 2007 and 2017 was used for comparison. Exclusion criteria for healthy volunteers were neurological or other major illness, pregnancy and intolerability to PET or MRI.

The study was conducted according to the guidelines of the Declaration of Helsinki. Study was approved by the Ethical Committee of the Hospital District of Southwest Finland and the Local Research Ethics Committee, as well as the Administration of Radioactive Substances Advisory Committee in the UK. Informed consent form was obtained from all subjects involved in the study.

PET imaging using the TSPO-radioligand [^11^C](R)-PK11195 was performed to evaluate TSPO-expressing microglia in people with MS and HCs. For anatomic reference and the evaluation of MS pathology, MRI was obtained at the time of PET imaging. Brain MRI was performed with Philips Gyroscan Intera 1.5T Nova Dual (*n* = 25) or 3T Ingenuity TF PET/MR (*n* = 114) scanners at TPC. At WMIC, a Philips Achieva 1.5T (*n* = 25) or 3T (*n* = 14) MR scanner was used. The protocol of brain MRI for HCs included at least a 3D T1-weighted sequence. For MS patients, a T2-weighted sequence, and a fluid-attenuated inversion recovery (FLAIR) sequence were also included for the evaluation of T2 lesions. In addition, MS patients were evaluated clinically for disability using EDSS at the time of imaging.

### 2.2 [^11^C](R)-PK11195 radioligand production and PET image acquisition

The radiochemical synthesis of [^11^C](R)-PK11195 was performed as described previously (9, 18). The mean [standard deviation (SD)] injected dose was 472 (28) MBq for MS patients and 553 (112) MBq for HCs (*p* < 0.001). In both centers, PET imaging was performed using a brain dedicated ECAT High-Resolution Research Tomograph scanner (CTI/Siemens) with an intrinsic spatial resolution of approximately 2.5 mm. A 6-min transmission scan for attenuation correction was obtained using a ^137^Cs point source. After that, a 60-min dynamic emission PET scan was started simultaneously with the intravenous bolus injection of the radioligand. A thermoplastic mask was used to minimize head movements at TPC.

### 2.3 Creation of region of interest masks

T2- and T1-lesion ROI masks were created based on MR images performed concurrently with TSPO-PET imaging. A semi-automated method by the Lesion Segmentation Tool (28) and Carimas with manual editing slice by slice was used as described previously (18). Freesurfer 5.3 software was used to segment cortical gray matter (cGM), white matter and thalamus after the T1-lesion filling as previously reported (9). The whole brain ROI included supratentorial brain parenchyma, cerebellum, and brain stem. The NAWM ROI was created by removing the T2 lesion ROI from the WM ROI.

### 2.4 PET post-processing and analysis

PET images were reconstructed using 17-time frames post tracer injection, and the dynamic data were then smoothed using a Gaussian 2.5-mm post-reconstruction filter as described previously (9, 18). Possible displacements between frames due to head motion were corrected using mutual information realignment in SPM8. Finally, PET images were co-registered with the 3D T1 MRI. TSPO-expressing microglia were evaluated as specific binding of [^11^C](R)-PK11195 using distribution volume ratio (DVR) in the prespecified ROIs: whole brain, NAWM, cGM, and thalamus. A supervised clustering technique was used to derive a clustered reference area, and ROI-level DVRs were estimated as previously described (18).

### 2.5 Statistical analysis

The statistical analyses were performed using R (version 4.2.1). Variables are reported as mean (SD) unless otherwise stated. For all analyses, a *p*-value <0.05 was considered statistically significant. MRI volumetric parameters are presented as a parenchymal fraction (PF), defined as the ratio of brain parenchymal volume to total intracranial volume unless otherwise stated.

DVR values of the prespecified ROIs were analyzed in HCs, all MS patients and in subgroups of RRMS and SPMS, as well as stratified by sex. Wilcoxon rank-sum test was used for group comparisons between categorical variables and DVRs. Multiple comparisons were corrected using a false discovery rate (FDR). Spearman correlation test was used to evaluate correlations of DVRs and EDSS. Reported *p*-values are naïve unless otherwise stated.

To adjust the results for confounding factors, a multiple linear regression model was used for patient data, and a linear mixed model with center as a random effect for controls, as they were from two different imaging centers. R software package lmerTest (version 3.1.3) was used for the mixed model. The used covariates were age, sex, brain volume (cm^3^) and body mass index (BMI). The model assumption was checked using Shapiro–Wilk’s test (for normality of the residuals), variance inflation factors and Cook’s distance (for influential observations), and, in case of influential observations, results were confirmed by analyzing the dataset without such observation.

Voxel-wise parametric binding potential (BP_ND_) maps were calculated using basis function implementation of SRTM (29) with 250 basis functions. These were then converted into DVR (BP_ND_ + 1). Lower and upper bounds for theta were set to 0.06 1/min and 0.8 1/min. The resulting parametric maps were normalized to MNI space (Montreal Neurological Institute database) in SPM8. Mean PET images were obtained using the normalized parametric maps. The associations of radioligand binding and EDSS were evaluated with multiple regression model using normalized parametric binding potential images in MNI space. Analysis was performed for all MS patients, as well as for male and female patients separately. To ensure normality before the statistical analysis, the images were smoothed with 3D Gaussian 8 mm FWHM (full width at half maximum) filter. Multiple comparisons were corrected at the cluster level using the FWE (family-wise error) rate, and the critical significance level to reject the null hypothesis was set to 0.001.

## 3 Results

### 3.1 Demographic and clinical characteristics of study participants

The study cohort consisted of 102 MS patients with a mean (SD) age of 45.3 (9.7) years most of whom were females (*n* = 73, 72%). Median disease duration at the time of PET imaging was 12.1 [interquartile range (IQR) 7.0–17.2] years, and median EDSS 3.0 (IQR 2.0–3.9). No significant differences in age, disease duration, EDSS, annualized relapse rate (ARR) from disease onset to PET imaging, treatment, or BMI were observed between male and female patients at PET imaging (Table 1). All demographic information and MS-disease-related details are presented in Table 1.

**Table 1:** Demographic and clinical characteristics of the study cohort.

	HC	MS	HC vs. MS, *p*-value	RRMS	SPMS	RRMS vs. SPMS, *p*-value	Male MS	Female MS	Male vs. female MS, *p*-value
Subjects, *n*	76	102		75	27		29	73	
Female, *n* (%)	46 (61)	73 (72)	0.15	56 (75)	17 (63)	0.3	–	–	–
Age (years)	45.5 (14.5)	45.3 (9.7)	0.8	42.9 (8.9)	51.7 (9.2)	**<0.001**	45.9 (9.8)	45.0 (9.8)	0.9
BMI (kg/m^2^)†	25.2 (4.0)	26.5 (5.8)	0.3	27.2 (5.8)	24.8 (5.8)	**0.024**	26.5 (5.3)	26.6 (6.1)	0.6
Disease duration (years), median (IQR)	–	12.1 (7.0–17.2)	–	9.6 (4.8–13.8)	19.2 (13.9–22.9)	**<0.001**	9.8 (3.2–16.9)	12.2 (7.7–17.3)	0.3
EDSS, median (IQR)	–	3.0 (2.0–3.9)	–	2.5 (2.0–3.0)	6.0 (4.25–6.5)	**<0.001**	3.0 (2.0–3.5)	3.0 (2.0–4.0)	0.9
ARR, median (IQR)	–	0.31 (0.22–0.53)	–	0.4 (0.2–0.6)	0.24 (0.18–0.40)	**0.010**	0.4 (0.2–0.6)	0.3 (0.2–0.5)	0.6
Disease modifying therapy, *n* (%)									
No therapy	–	48 (47)	–	25 (33)	23 (85)	**< 0.001**	14 (48)	34 (47)	0.3
Low-moderate efficacy	–	51 (50)	–	47 (63)	4 (15)		13 (45)	38 (52)	
High-efficacy	–	3 (3)	–	3 (4)	0 (0)		2 (7)	1 (1)	

Values are mean (SD) unless otherwise stated. For group comparisons, value of *p*s are from Wilcoxon rank-sum test. Significant *p*-values are bolded. Disease duration is years from first symptoms. Low-moderate efficacy therapy includes interferon beta, glatiramer acetate, dimethyl fumarate, teriflunomide and fingolimod. High-efficacy therapy includes natalizumab and rituximab. ^†^BMI has one missing value in MS (female, RRMS) and two missing values in HC (male and female). ARR, annualized relapse rate; BMI, body mass index; EDSS, Expanded Disability Status Scale; HC, healthy control; MS, multiple sclerosis; RRMS, relapsing remitting MS; SPMS, secondary progressive MS.

### 3.2 Brain MRI outcomes of the study participants

Brain volume, NAWM, cortical GM (cGM) and thalamus volumes were larger among HCs compared to patients with MS (Table 2). As expected, SPMS patients had smaller brain volume as well as smaller NAWM and cGM volumes, but larger T1 and T2 lesion loads compared to RRMS patients (Table 2). Among all the studied subgroups, i.e., all MS, HCs, RRMS and SPMS subjects, the total brain volume (cm^3^) was larger in males compared to females. When brain volume was normalized to the total intracranial volume (PF), healthy females had larger brain volume than female MS-patients, but no such difference was observed in males (Table 3). Of the subregions of interest, male RRMS patients had larger NAWM volumes compared to females. No differences in the T1 or T2 lesion loads between male and female RRMS patients was observed. Among SPMS patients, no differences in brain volumes between males and females were observed. The NAWM, cGM and thalamus volumes were larger among healthy females compared to female MS patients. Volumetric differences were less prominent between male patients and control males, where cGM and thalamus, but not NAWM, volumes were larger among HC males (Table 3).

**Table 2:** MRI volumetric parameters of healthy controls, all MS patients and RRMS- and SPMS-subgroups.

	HC	MS	HC vs. MS, *p*-value	RRMS	SPMS	RRMS versus SPMS
Brain volume (PF)	0.855 (0.036)	0.828 (0.046)	**<0.001**	0.834 (0.043)	0.810 (0.050)	**0.034**
NAWM volume (PF)	0.35 (0.025)	0.33 (0.036)	**<0.001**	0.33 (0.026)	0.32 (0.053)	**0.035**
Cortical GM volume (PF)	0.33 (0.022)	0.31 (0.025)	**<0.001**	0.32 (0.021)	0.30 (0.031)	**0.003**
Thalamus volume (PF)	0.011 (0.0011)	0.010 (0.0013)	**<0.001**	0.010 (0.0011)	0.010 (0.0017)	0.059
T1 lesion load (cm^3^), median (IQR)	–	2.95 (1.28–8.58)	–	2.32 (1.11–4.17)	11.2 (6.95–23.2)	**<0.001**
T2 lesion load (cm^3^), median (IQR)	–	6.01 (2.88–17.6)	–	4.14 (2.60–10.9)	18.5 (11.1–34.6)	**<0.001**

Values are mean (SD) unless otherwise stated. For group comparisons, *p*-values are from Wilcoxon rank-sum test. Significant *p*-values are bolded. GM, gray matter; HC, healthy control; IQR, interquartile range; MS, multiple sclerosis; NAWM, normal-appearing white matter; PF, parenchymal fraction; RRMS, relapsing remitting MS; SPMS, secondary progressive MS.

**Table 3:** MRI volumetric parameters of the study cohort by gender.

	All MS patients			RRMS patients			SPMS patients			HCs				
	Males	Females	*p*-value	Males	Females	*p*-value	Males	Females	*p*-value	Males	Females	*p*-value	Males HC versus MS, *p*-value	Females HC versus MS, *p*-value
Brain volume (PF)	0.83 (0.06)	0.83 (0.04)	0.3	0.85 (0.05)	0.83 (0.04)	0.063	0.81 (0.06)	0.81 (0.04)	0.9	0.86 (0.03)	0.85 (0.04)	0.6	0.2	**<0.001**
Brain volume (cm^3^)	1.228 (122)	1.113 (95)	**<0.001**	1.265 (120)	1.128 (92)	**<0.001**	1.157 (92)	1.062 (89)	**0.027**	1.233 (99)	1.118 (105)	**<0.001**	0.6	0.7
NAWM volume (PF)	0.33 (0.043)	0.33 (0.033)	0.5	0.35 (0.027)	0.33 (0.027)	**0.030**	0.31 (0.057)	0.32 (0.052)	0.3	0.35 (0.024)	0.35 (0.026)	0.17	0.051	**0.003**
Cortical GM volume (PF)	0.31 (0.030)	0.31 (0.022)	0.4	0.32 (0.025)	0.32 (0.019)	0.8	0.30 (0.037)	0.30 (0.027)	0.2	0.33 (0.018)	0.33 (0.024)	0.5	**0.006**	**<0.001**
Thalamus volume (PF)	0.010 (0.0013)	0.010 (0.0013)	0.3	0.010 (0.0010)	0.010 (0.0011)	0.9	0.009 (0.0013)	0.010 (0.0019)	0.3	0.0105 (0.0009)	0.0112 (0.0012)	**0.015**	**0.041**	**<0.001**
T1 lesion load (cm^3^), median (IQR)	2.78 (1.39–11.2)	3.28 (1.17–8.20)	0.6	2.04 (1.28–2.84)	2.33 (0.79–5.02)	0.7	13.88 (8.27–20.7)	8.71 (4.57–25.0)	0.4	–	–	–	–	–
T2 lesion load (cm^3^), median (IQR)	10.7 (3.62–16.5)	5.43 (2.67–17.8)	0.2	4.19 (3.32–11.04)	4.07 (2.34–10.8)	0.4	20.8 (15.3–40.9)	17.8 (7.64–33.4)	0.6	–	–	–	–	–

Values are mean (SD) unless otherwise stated. For group comparisons, value of ps are from Wilcoxon rank-sum test. Significant *p*-values are bolded. GM, gray matter; IQR, interquartile range; MS, multiple sclerosis; NAWM, normal-appearing white matter; PF, parenchymal fraction; RRMS, relapsing remitting MS; SPMS, secondary progressive MS.

### 3.3 TSPO radioligand binding in patients with MS vs. healthy controls and in RRMS vs. SPMS

**Figure 1:**
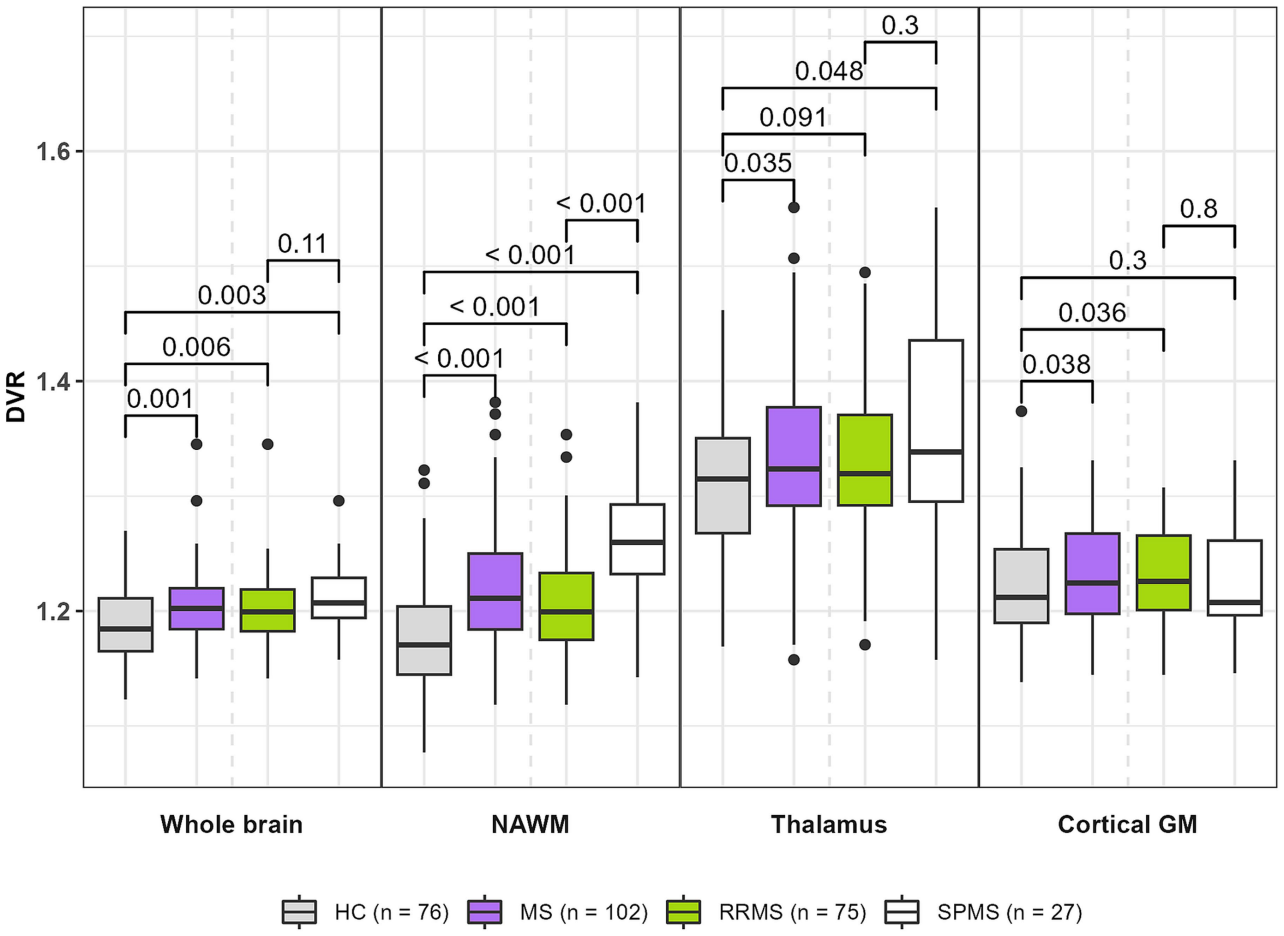
TSPO-PET DVR values in MS patients (all MS, RRMS, and SPMS) and healthy controls. MS patients had higher DVRs in whole brain, NAWM, thalamus and cortical GM compared to healthy controls. SPMS patients had a higher DVR in NAWM compared to RRMS patients. Compared to HCs, RRMS patients had higher DVRs in whole brain, NAWM and cortical GM. Compared to HCs, SPMS patients had higher DVRs in whole brain, NAWM and thalamus. Wilcoxon rank-sum test was used. All significant results in whole brain and NAWM also survived the FDR-correction, but not in thalamus and cortical GM. DVR, distribution volume ratio; GM, gray matter; HC, healthy control; MS, multiple sclerosis; NAWM, normal-appearing white matter; PET, positron emission tomography; RRMS, relapsing remitting MS; SPMS, secondary progressive MS; TSPO, translocator protein.

[^11^C](R)-PK11195 DVR values were higher in patients with MS compared to HCs in the whole brain [1.20 (0.03) vs. 1.19 (0.03), *p* = 0.001], NAWM [1.22 (0.05) vs. 1.18 (0.05), *p* < 0.001], thalamus [1.34 (0.08) vs. 1.31 (0.07), *p* = 0.035] and cGM [1.23 (0.04) vs. 1.22 (0.05), *p* = 0.038] (Figure 1). SPMS patients had a higher DVR in the NAWM compared to RRMS patients [1.26 (0.06) vs. 1.20 (0.05), *p* < 0.001]. No difference in TSPO-binding in other ROIs was observed between SPMS patients and RRMS patients. RRMS patients and SPMS patients had higher DVRs in whole brain, NAWM and thalamus compared to HCs. All significant results in whole brain and NAWM also survived the FDR correction, but not in the thalamus and cGM.

### 3.4 Association between TSPO radioligand binding and disability status in patients with MS

**Figure 2:**
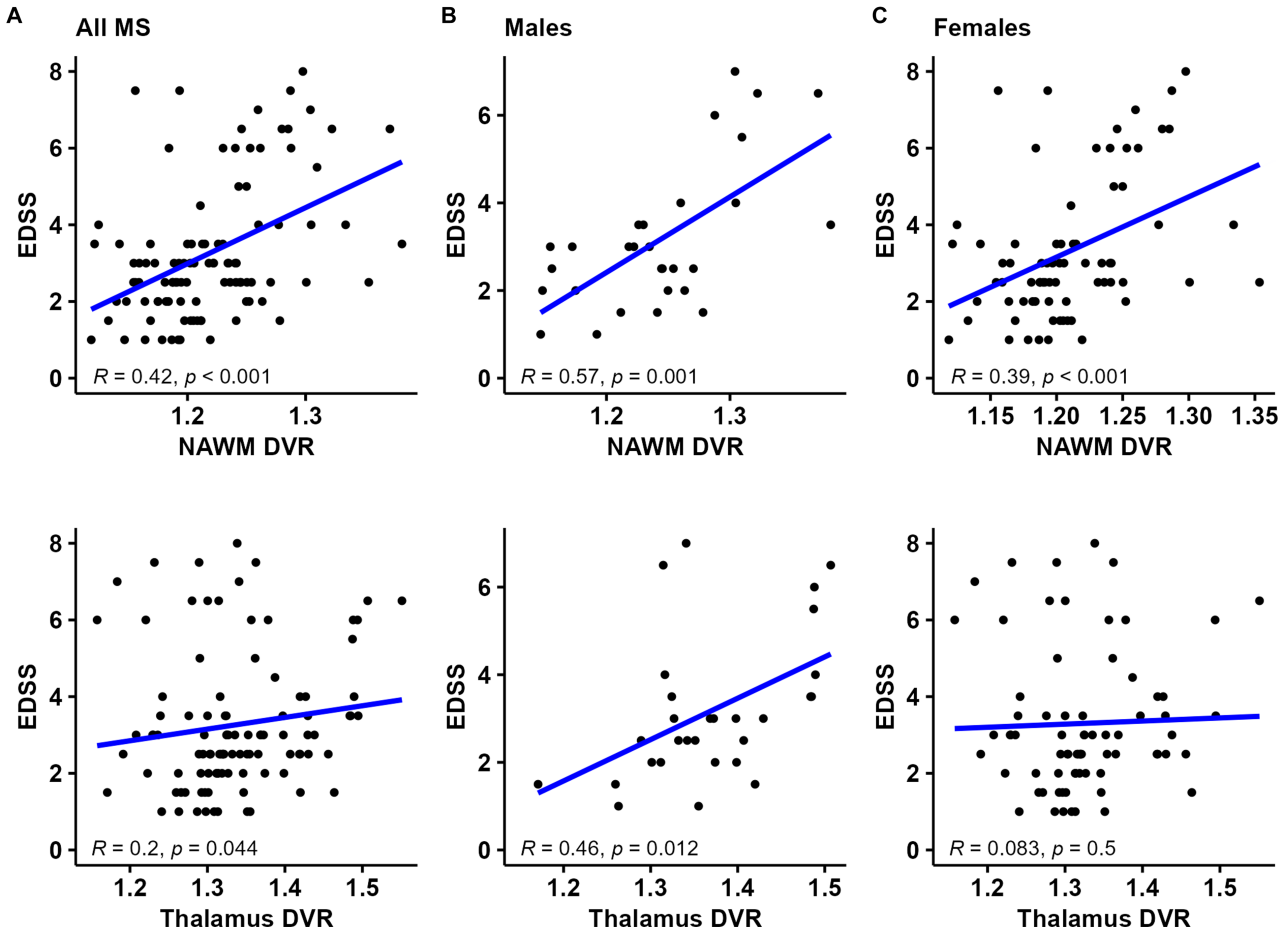
TSPO-PET DVR values and correlation with disability status in **(A)** all MS patients, **(B)** male MS patients, and **(C)** female MS patients. NAWM and thalamus DVR correlated with EDSS in all MS patients. The strength of correlation between NAWM DVR and EDSS was stronger among male patients compared to females. Correlation between thalamus DVR and EDSS was observed only in male MS patients. Spearman correlation test was used. DVR, distribution volume ratio; EDSS, Expanded Disability Status Scale; NAWM, normal-appearing white matter; PET, positron emission tomography; R, Pearson correlation Coefficient; TSPO, translocator protein.

**Figure 3:**
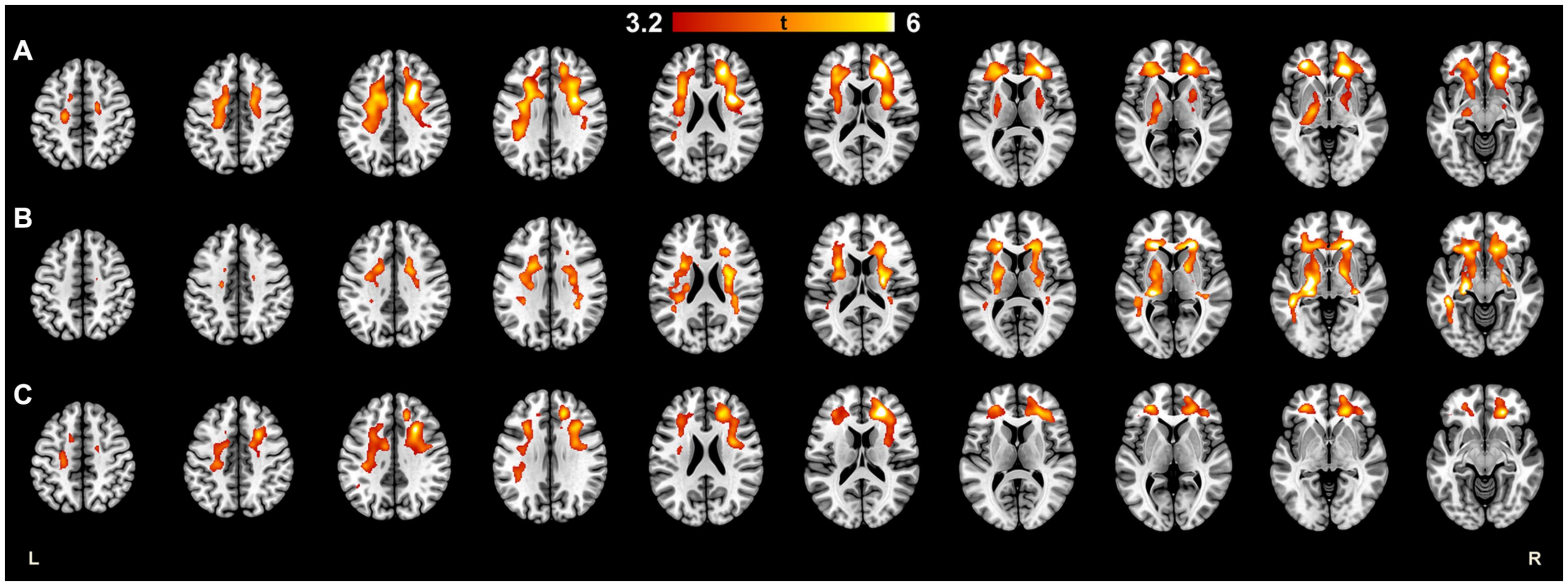
Statistical parametric mapping image demonstrating brain areas where the higher TSPO-radioligand binding was significantly associated with higher EDSS scores in **(A)** all MS patients, **(B)** male patients, and **(C)** female patients. High TSPO-binding in subcortical WM, especially in frontal WM was associated with EDSS in females and males. In male patients, high TSPO-binding in WM tract surrounding basal ganglia and thalamus was associated with EDSS. Multiple regression with EDSS as an explaining variable was performed using BPND images in MNI space. To ensure normality before the statistical analysis, the images were smoothed with 3D Gaussian 8 mm FWHM filter. Multiple comparisons were corrected using the FWE rate at cluster level, and the critical significance level to reject the null hypothesis was set to 0.001. The color scale of the marked areas refer to their t-statistic. BPND, normalized parametric binding potential; EDSS, Expanded disability status score; FWE, family-wise error; FWHM, full width at half maximum; HC, healthy control; MNI, Montreal Neurological Institute database; TSPO, translocator protein.

In all MS patients, a moderate correlation between NAWM DVR and EDSS and a weak correlation between thalamus DVR and EDSS was observed (*R* = 0.42, *p* < 0.001 and *R* = 0.2, *p* = 0.044; respectively) (Figure 2A). The strength of correlation between NAWM DVR and EDSS was higher among male patients compared to females (*R* = 0.57 vs. *R* = 0.39). Correlation between thalamus DVR and EDSS was observed only in male MS patients (Figures 2B,C). The association of TSPO-binding and EDSS was also evaluated using parametric binding potential images to demonstrate visually the brain areas with high TSPO-binding associated to EDSS. High TSPO-binding in subcortical WM, especially in frontal WM was associated with EDSS in both sexes. Furthermore, in male patients, high TSPO-binding in WM tract surrounding basal ganglia and thalamus was associated with EDSS (Figures 3A–C).

### 3.5 TSPO radioligand binding in males vs. females

**Figure 4:**
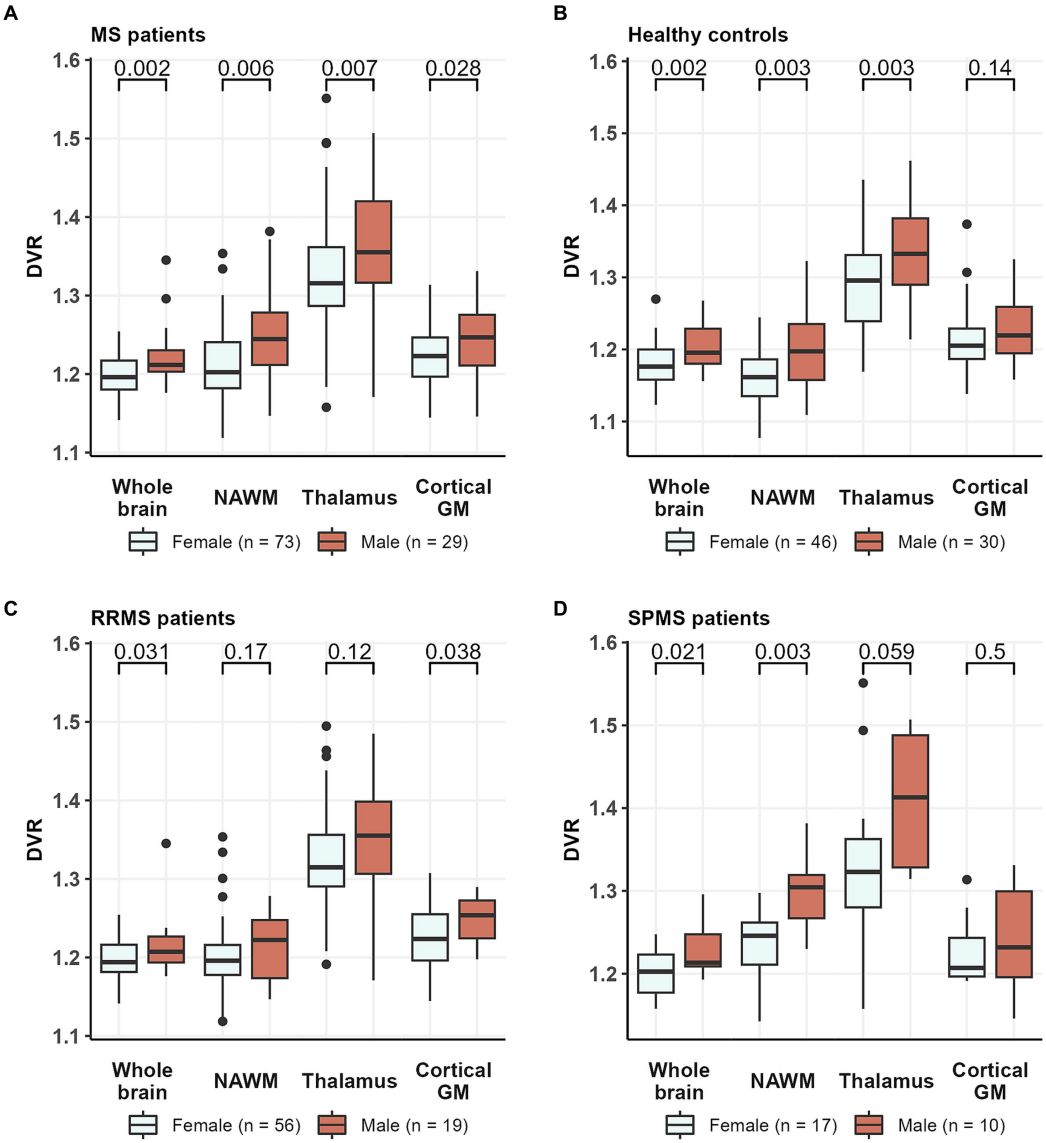
TSPO-PET DVR values in **(A)** all MS patients, **(B)** healthy controls, **(C)** RRMS patients, and **(D)** SPMS patients by sex. Male MS patients had a higher DVR in whole brain, NAWM, thalamus and cortical GM compared to female MS patients. Similarly, compared to healthy females, healthy males had a higher DVR in whole brain, NAWM and thalamus but not in cortical GM. Among RRMS patients, male patients had a higher DVR than females in whole brain and cortical GM, while in the SPMS group, male patients had a higher DVR than females in whole brain and NAWM. Wilcoxon rank-sum test was used. DVR, distribution volume ratio; MS, multiple sclerosis; NAWM, normal-appearing white matter; PET, positron emission tomography; RRMS, relapsing remitting multiple sclerosis; SPMS, secondary progressive multiple sclerosis; TSPO, translocator protein.

The specific binding of [^11^C](R)-PK11195 in the brain was increased in both MS patients and in healthy control males compared to females. Compared to female MS patients, male MS patients had a higher DVR in the whole brain [1.22 (0.04) vs. 1.20 (0.02), *p* = 0.002] and in the NAWM [1.24 (0.06) vs. 1.21 (0.05), *p* = 0.006], thalamus [1.37 (0.08) vs. 1.32 (0.02), *p* = 0.008] and cGM [1.25 (0.04) vs. 1.23 (0.04), *p* = 0.028] (Figure 4A). Similarly, compared to healthy females, healthy males had a higher DVR in the whole brain [1.20 (0.03) vs. 1.18 (0.03), *p* = 0.002], WM [1.20 (0.05) vs. 1.16 (0.04), *p* = 0.003], and thalamus [1.34 (0.06) vs. 1.29 (0.07), *p* = 0.003] but not in cGM [1.23 (0.04) vs. 1.21 (0.05), *p* = 0.14] (Figure 4B).

When RRMS- and SPMS-subgroups were analyzed separately, we noticed that sex differences in [^11^C](R)-PK11195 binding were more prominent among SPMS patients compared to RRMS. Among the RRMS patients, males had higher [^11^C](R)-PK11195-binding in the whole brain [1.22 (0.04) vs. 1.20 (0.02), *p* = 0.031] and cGM [1.25 (0.03) vs. 1.23 (0.04), *p* = 0.038], but not in the NAWM [1.21 (0.04) vs. 1.20 (0.05), *p* = 0.17] or thalamus [1.35 (0.07) vs. 1.32 (0.07), *p* = 0.12] (Figure 4C). Within the SPMS-subgroup, males had similarly a higher DVR in the whole brain [1.23 (0.03) vs. 1.20 (0.03), *p* = 0.021] but also in the NAWM [1.30 (0.05) vs. 1.24 (0.05), *p* = 0.003] compared to females. No statistically significant difference in [^11^C](R)-PK11195-binding was observed in the thalamus [1.41 (0.09) vs. 1.32 (0.10), *p* = 0.059] or cGM [1.24 (0.06) vs. 1.22 (0.04), *p* = 0.5] (Figure 4D).

**Figure 5:**
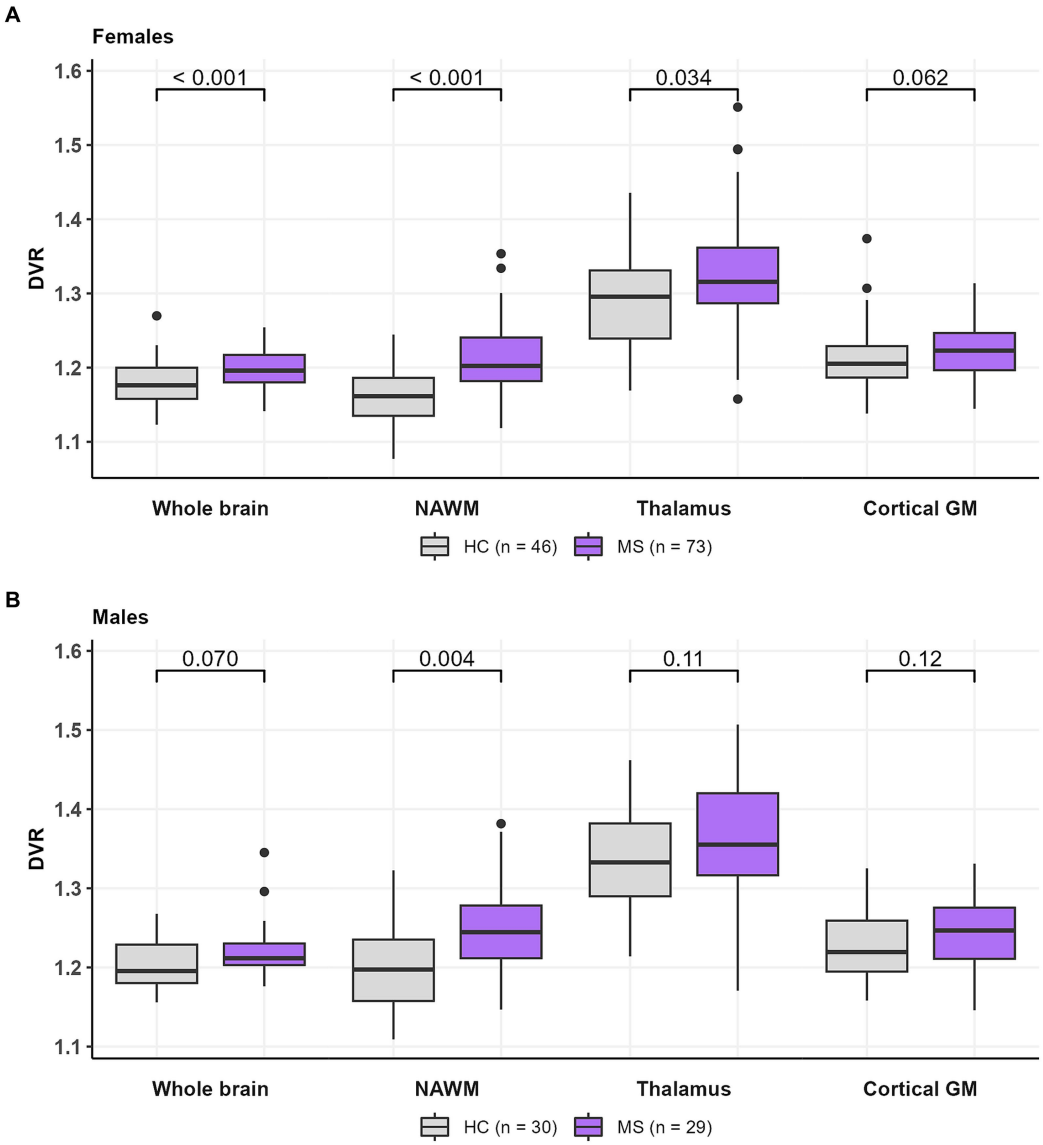
TSPO-PET DVR-values in **(A)** female HCs and MS patients and **(B)** male HCs and MS patients. Female MS patients had a higher DVR in whole brain, NAWM and thalamus compared to healthy females. Male MS patients had a higher DVR in NAWM compared to healthy males. No difference in DVR values in cortical GM between females and males were observed in HCs or MS patients. Wilcoxon rank-sum test was used. DVR, distribution volume ratio; HC, healthy control; MS, multiple sclerosis; NAWM, normal-appearing white matter; PET, positron emission tomography; TSPO, translocator protein.

Compared to healthy females, female MS patients had a higher DVR in the whole brain [1.20 (0.02) vs. 1.18 (0.03), *p* < 0.001], NAWM [1.21 (0.05) vs. 1.16 (0.04), *p* < 0.001] and thalamus [1.32 (0.08) vs. 1.29 (0.07), *p* = 0.034] (Figure 5A) but not in cGM [1.23 (0.04) vs. 1.21 (0.05), *p* = 0.062]. On the other hand, compared to healthy males, male MS patients had a higher DVR only in the NAWM [1.24 (0.06) vs. 1.20 (0.05), *p* = 0.004]. No significant difference in TSPO-binding was observed in the whole brain [1.22 (0.04) vs. 1.20 (0.03), *p* = 0.070], thalamus [1.37 (0.08) vs. 1.34 (0.06), *p* = 0.11] or cGM [1.25 (0.04) vs. 1.23 (0.04), *p* = 0.12] between male MS patients and HCs (Figure 5B).

Mean parametric PET-images demonstrating visually the differences in TSPO-binding in different subgroups of the study cohort are presented in Supplementary Presentation S1.

### 3.6 Adjustment of the [^11^C](R)-PK11195-binding results for confounding factors

When age, BMI and brain volume were considered as confounding factors in multiple linear regression modeling, the differences in DVR values between female and male MS patients as well as in healthy males and females remained statistically significant in the whole brain (*p* = 0.006 in MS group and 0.03 in HC group), NAWM (*p* < 0.001 in MS group and p = 0.006 in HC group) and thalamus (*p* = 0.006 in MS group and *p* = 0.007 in HC group) (Table 4). Among RRMS- and SPMS-subgroups, only the sex difference in SPMS NAWM DVR (*p* = 0.004) remained significant after adjusting for confounding factors. In addition, after adjusting for confounding factors, male SPMS patients had a higher DVR in the thalamus (*p* = 0.040) compared to females, although initially, there was no sex difference in thalamic DVR in the SPMS-subgroup.

**Table 4:** Modeling the impact of sex and confounding factors on DVR in various brain regions in the studied cohorts.

Healthy controls (*n* = 76)					
**Outcome DVR**	**Predictors**	**Estimate**	**95% CL**		***p*-value**
NAWM	Male vs. female	0.036	0.010	0.061	**0.006**
	Age	0.0008	−0.0001	0.0016	0.071
	Brain volume	0.0001	−0.0001	0.0002	0.3
	BMI	0.0001	−0.0025	0.0027	0.9
Whole brain	Male vs. female	0.018	0.002	0.033	**0.030**
	Age	0.0004	−0.0002	0.0009	0.19
	Brain volume	0.0000	0.0000	0.0001	0.2
	BMI	0.0018	0.0001	0.0034	**0.042**
Thalamus	Male vs. female	0.049	0.014	0.084	**0.007**
	Age	−0.0001	−0.0013	0.0010	0.8
	Brain volume	0.0000	−0.0001	0.0002	0.9
	BMI	−0.0024	−0.0061	0.0013	0.2
Cortical GM	Male vs. female	0.008	−0.017	0.033	0.5
	Age	0.0001	−0.0007	0.0010	0.8
	Brain volume	0.0000	−0.0001	0.0002	0.5
	BMI	0.0030	0.0003	0.0057	**0.027**

MS cohort (*n* = 102)					
**Outcome DVR**	**Predictors**	**Estimate**	**95% CL**		***p*-value**
NAWM	Male vs. female	0.046	0.021	0.070	**<0.001**
	Age	0.0008	−0.0003	0.0018	0.17
	Brain volume	−0.0001	−0.0002	0.0000	**0.047**
	BMI	−0.0021	−0.0038	−0.0004	**0.015**
Whole brain	Male vs. female	0.020	0.006	0.034	**0.006**
	Age	0.0003	−0.0003	0.0009	0.3
	Brain volume	0.0000	0.0000	0.0001	0.6
	BMI	0.0007	−0.0002	0.0017	0.14
Thalamus	Male vs. female	0.055	0.016	0.094	**0.006**
	Age	−0.0005	−0.0022	0.0012	0.5
	Brain volume	−0.0001	−0.0002	0.0001	0.3
	BMI	−0.0027	−0.0053	0.0000	**0.047**
Cortical GM	Male vs. female	0.007	−0.012	0.026	0.4
	Age	0.0009	0.0000	0.0017	**0.039**
	Brain volume	0.0001	0.0000	0.0002	**0.005**
	BMI	0.0015	0.0001	0.0028	**0.033**

SPMS cohort (*n* = 27)					
**Outcome DVR**	**Predictors**	**Estimate**	**95% CL**		***p*-value**
NAWM	Male vs. female	0.069	0.024	0.114	**0.004**
	Age	−0.0017	−0.0041	0.0007	0.15
	Brain volume	0.0000	−0.0003	0.0002	0.8
	BMI	0.0014	−0.0025	0.0053	0.5
Whole brain	Male vs. female	0.027	−0.001	0.054	0.058
	Age	0.0003	−0.0012	0.0018	0.7
	Brain volume	0.0001	−0.0001	0.0002	0.4
	BMI	0.0015	−0.0008	0.0039	0.19
Thalamus	Male vs. female	0.087	0.004	0.169	**0.040**
	Age	−0.0045	−0.0089	−0.0001	**0.046**
	Brain volume	−0.0001	−0.0005	0.0003	0.6
	BMI	−0.0025	−0.0096	0.0046	0.5
Cortical GM	Male vs. female	0.008	−0.029	0.044	0.7
	Age	0.0016	−0.0003	0.0036	0.093
	Brain volume	0.0002	0.0000	0.0004	**0.028**
	BMI	0.0028	−0.0003	0.0059	0.078

RRMS cohort (*n* = 75)					
**Outcome DVR**	**Predictors**	**Estimate**	**95% CL**		***p*-value**
NAWM	Male vs. female	0.021	−0.007	0.049	0.14
	Age	0.0005	−0.0007	0.0018	0.4
	Brain volume	−0.0001	−0.0002	0.0001	0.4
	BMI	−0.0017	−0.0035	0.0001	0.068
Whole brain	Male vs. female	0.014	−0.003	0.032	0.11
	Age	0.0001	−0.0007	0.0008	0.9
	Brain volume	0.0000	0.0000	0.0001	0.5
	BMI	0.0007	−0.0004	0.0019	0.2
Thalamus	Male vs. female	0.030	−0.014	0.075	0.18
	Age	0.0008	−0.0011	0.0028	0.4
	Brain volume	0.0000	−0.0002	0.0001	0.6
	BMI	−0.0013	−0.0042	0.0015	0.4
Cortical GM	Male vs. female	0.008	−0.015	0.031	0.5
	Age	0.0004	−0.0006	0.0015	0.4
	Brain volume	0.0001	0.0000	0.0002	0.054
	BMI	0.0010	−0.0006	0.0026	0.2

Modeling of the data was performed to explore the impact of sex with various confounding factors on DVR values in various brain regions in all studied cohorts. Results are based on linear mixed model (HCs) and multiple linear regression models (whole MS cohort and its subcohorts). Estimates with 95% confidence intervals and *p*-values for all predictors are presented in each model. In all other cohorts but in RRMS the impact of sex on TSPO-expressing microglia remained significant in most brain areas when confounding factors were considered. Significant *p*-values are bolded. The model assumption was checked and in case of an influential observations and results were confirmed by analyzing the dataset without such observation. This was the case in whole MS cohort with whole brain DVR as an outcome, in RRMS cohort with NAWM and whole brain DVRs as outcomes, and in SPMS cohort with cortical GM as an outcome. However, inferences regarding sex differences did not change. BMI, body mass index; CL, confidence interval; DVR, distribution volume ratio; GM, gray matter; HC, healthy control; MS, multiple sclerosis; NAWM, normal-appearing white matter; RRMS, relapsing–remitting MS; SPMS, secondary progressive MS.

## 4 Discussion

We sought to uncover differences in MS brain pathology among males and females that might contribute to a greater male propensity for clinical disability progression. Using TSPO-PET-imaging, we found increased TSPO-binding in several regions of interest in male MS brain compared to female MS brain. Interestingly, in healthy controls, TSPO-binding was similarly more prominent among males compared to females. This suggests that the TSPO-binding cells possess a sex-driven inherent variability, which may predispose male MS patients to a greater likelihood of progressive disease. The same phenomenon may contribute to the higher male prevalence and incidence of other neurodegenerative diseases, such as Parkinson’s disease and amyotrophic lateral sclerosis (30, 31). Hence, a better understanding of sex differences concerning microglia and macrophage responses may contribute to better prevention of progression, and more successful treatment outcomes.

In addition to evaluating TSPO-binding between males and females among MS patients and healthy controls, we also assessed variability in TSPO-binding among SPMS patients and RRMS patients according to sex. We found that among the entire cohort of MS patients and healthy controls, male SPMS patients had the highest TSPO availability, whereas healthy females had the lowest TSPO availability. This agrees with association of microglial activation with neurodegeneration (32), a feature most prominent among SPMS males.

Microglia are myeloid cells that reside in the CNS and comprise 5 to 10% of total cells in the brain, depending on the region examined (33). As non-neuronal cells, microglia originate from common myeloid precursor cells in the embryonic yolk sac early in ontogeny (34, 35). Microglia have a critical role during brain development and brain masculinization (35, 36). In mouse studies, the number and phenotype of microglia differ between males and females regionally, and in a time-specific manner (37, 38). There are mouse studies suggesting that adult microglia harbor several sex-biased features in their endogenous functions and responses to exogenous stimuli (39). Microglia from male mice express more genes associated with inflammatory processes, including regulation of cell migration and cytokine production, whereas female microglia express more genes linked to inhibition of inflammatory responses and promotion of repair mechanisms (40). Male microglia showed higher *in vitro* migration capacity than female microglia, both under basal and pro-inflammatory conditions, but female microglia had higher basal and pro-inflammatory stimulated phagocytic activity than male microglia (41).

Besides altered microglial phenotype, higher TSPO-binding may also signify higher microglial *density* (12). Microglial density in the brain is shown to be region dependent. For example, mouse studies have demonstrated significantly higher Iba1^+^ microglial cell density in the hippocampus, cortex, and amygdala in adult males compared to adult females (37). Microglia are long-lived cells which slowly renew throughout life (42). It is not presently known whether increased recruitment of microglial progenitor cells in early development or more microglial proliferation and/or less microglial apoptosis throughout male life might explain the increased microglial density in males (36, 43). As with the previously discussed mouse studies, it is plausible that the higher TSPO availability in male MS and control brains reflects higher glial density in addition to altered phenotype among males compared to females. Interestingly, the TSPO molecule has been ascribed both proliferative and anti-apoptotic actions (44). To date, we are not aware of neuropathological studies in MS or in healthy individuals addressing this question. Given the sex differences in TSPO-binding in HCs, it is important to take this aspect into consideration with careful sex-matching of control cohorts in all future TSPO-PET and MRI studies addressing smoldering inflammation.

Differential signaling by steroid hormones may explain variations between male and female microglia as microglia express hormone receptors. Microglia from adult mouse brain express one or both estrogen receptor (ER) subtypes (ERα, ERβ) (45, 46). In several studies, estrogen has shown to have an anti-inflammatory effect on microglia (47). Treatment of rat microglial cells with estrogen results in reduced lipopolysaccharide (LPS) -induced production of proinflammatory molecules such as inducible nitric oxide with reactive oxygen species production and prostaglandin E2 (48). Selective estrogen-receptor modulators have been shown to suppress the production of proinflammatory cytokines and chemokines after LPS stimulation *in vitro* (49). Ovariectomy in rodents associates with changes in microglia morphology and accumulation of mRNA encoding inflammatory mediators (50). In the experimental autoimmune encephalomyelitis (EAE) estrogen alleviates EAE severity both in males and in females (51). The more pronounced TSPO-binding observed in our study among healthy men and male MS patients could possibly be explained by the more prominent protective role of estrogen in females.

Sex differences in microglial activation patterns following other brain injuries have been reported, but mostly using experimental animal models. In experimental stroke models, expression of pro-inflammatory markers by microglia were shown to be higher in males compared to females (52, 53). Microglia and infiltrating myeloid cells also have an important role in neuroinflammatory responses following traumatic brain injury (TBI). Male mice showed higher Iba-1-positive microglial density at the post-traumatic lesion border in the cortex (54), but the data regarding sex differences in TBI remain somewhat conflicting (55, 56). A study addressing the sex difference in binding of a second generation TSPO-ligand, [^11^C]PBR28, gave conflicting results regarding the above-described male predominance in TSPO-binding. Interestingly, this effect was abolished with aging (57). Altogether, limited number of studies investigating sex differences in TSPO radioligand binding are available and therefore further studies addressing this question are needed.

The sex difference in TSPO-binding described in this study extends to other myeloid cells, particularly macrophages. In a mouse obesity study, male macrophages were more inflammatory and more migratory compared to female macrophages, which had higher expression of anti-inflammatory cytokines (58). In another obesity study, male mice increased their myelopoiesis which enhanced the inflammatory phenomena in adipose tissue. In a competitive bone marrow transplant experiment, this took place irrespective of the hormonal status but was dependent on intrinsic sex differences in the myeloid cells (59). Similarly, in our cohort, we observed no statistically significant difference in TSPO-binding between pre-and post-menopausal women (data not shown), which together with the above-described rodent experiments suggests that the sex difference in TSPO binding might not be entirely driven by estrogens.

## 5 Conclusion

In conclusion, our work demonstrates for the first time increased TSPO-binding in several brain regions in males vs. females, both among people with MS and in healthy controls. This implies fundamental differences in innate immune cell phenotype and function between the sexes. Given the well-appreciated role of smoldering inflammation as a driver of MS progression, our work gives a plausible explanation for the increased risk of progression and neurodegeneration among men with MS.

## Data availability statement

The raw data supporting the conclusions of this article will be made available by the authors, without undue reservation.

## Ethics statement

The study was approved by Ethical committee of the Hospital District of Southwest Finland, Local Research Ethics Committee and Administration of Radioactive Substances Advisory Committee in the UK. The studies were conducted in accordance with the local legislation and institutional requirements. The participants provided their written informed consent to participate in this study.

## Author contributions

SL: Conceptualization, Data curation, Investigation, Writing – original draft, Writing – review & editing. MS: Conceptualization, Writing – original draft, Writing – review & editing. MN: Investigation, Resources, Writing – review & editing. RH: Investigation, Resources, Writing – review & editing. AS: Investigation, Resources, Writing – review & editing. JR: Resources, Writing – review & editing. MM: Data curation, Formal analysis, Methodology, Writing – review & editing. LA: Conceptualization, Funding acquisition, Methodology, Supervision, Writing – original draft, Writing – review & editing.
