# Supplementary figures and images for: Sex-driven variability in TSPO-expressing microglia in MS patients and healthy individuals
Source: Front Neurol. 2024 Feb 20;15:1352116. doi: 10.3389/fneur.2024.1352116 (PMC10913932; doi:10.3389/fneur.2024.1352116)

## Slide 1
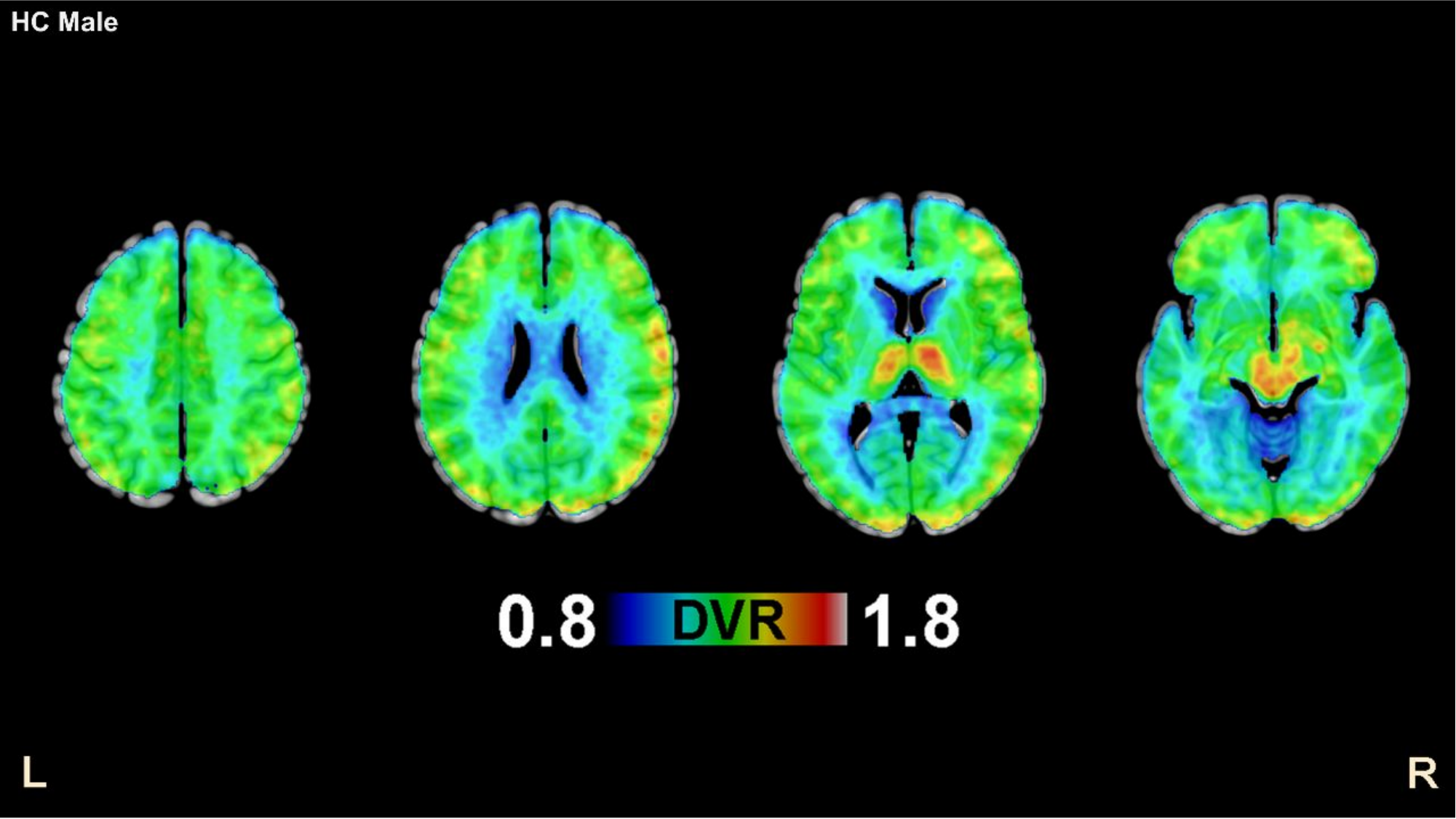

## Slide 2
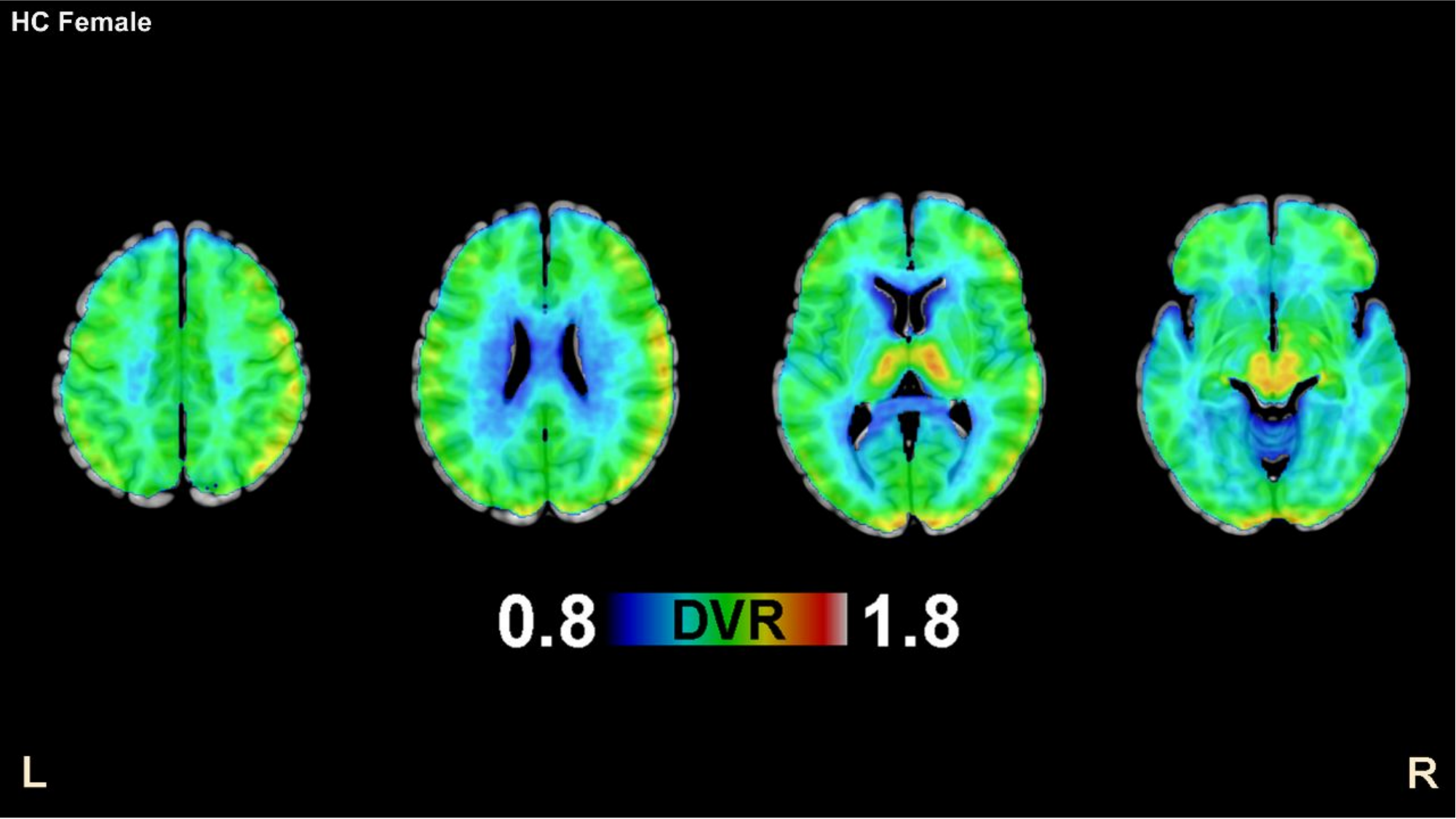

## Slide 3
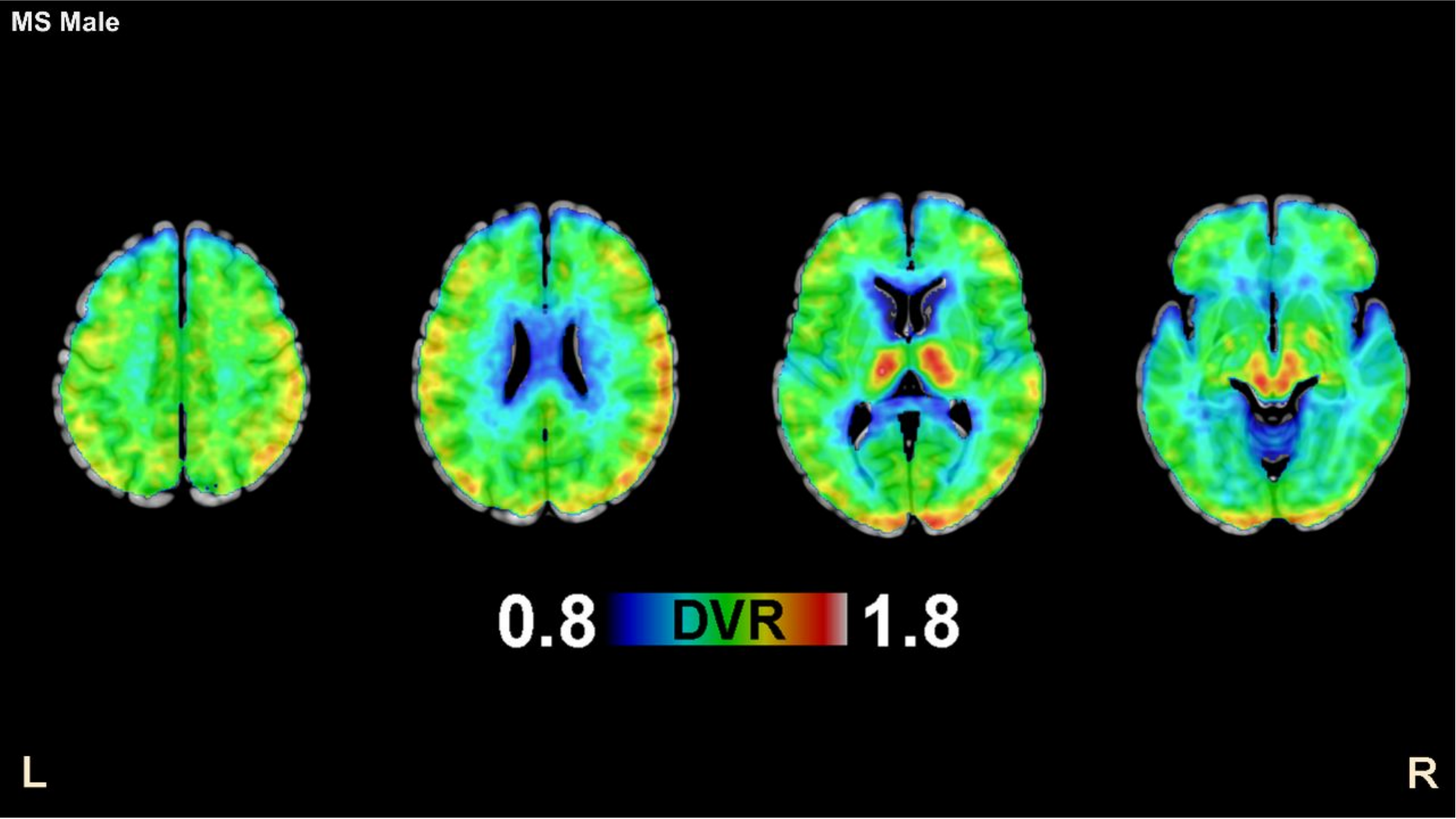

## Slide 4
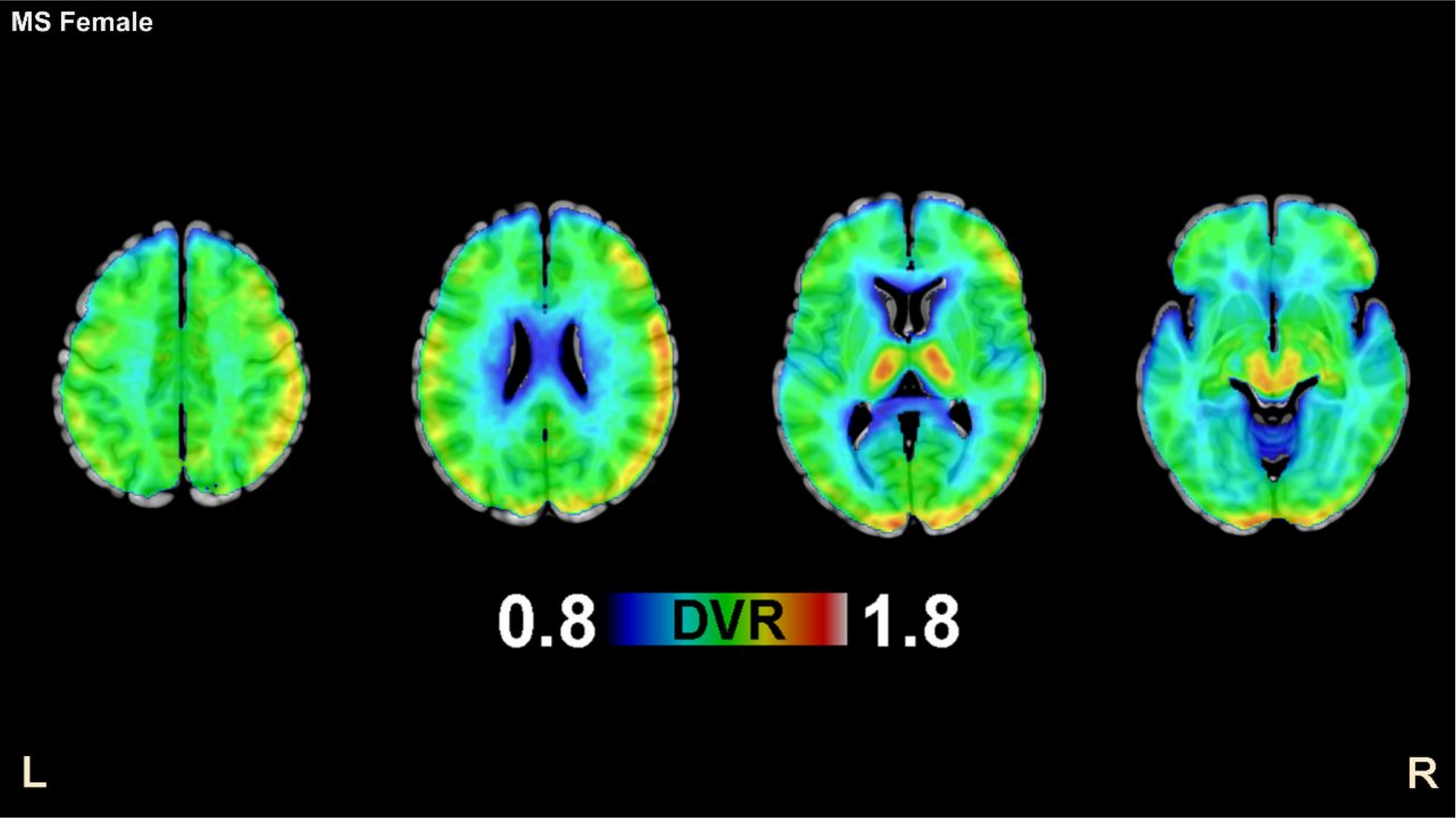

## Slide 5
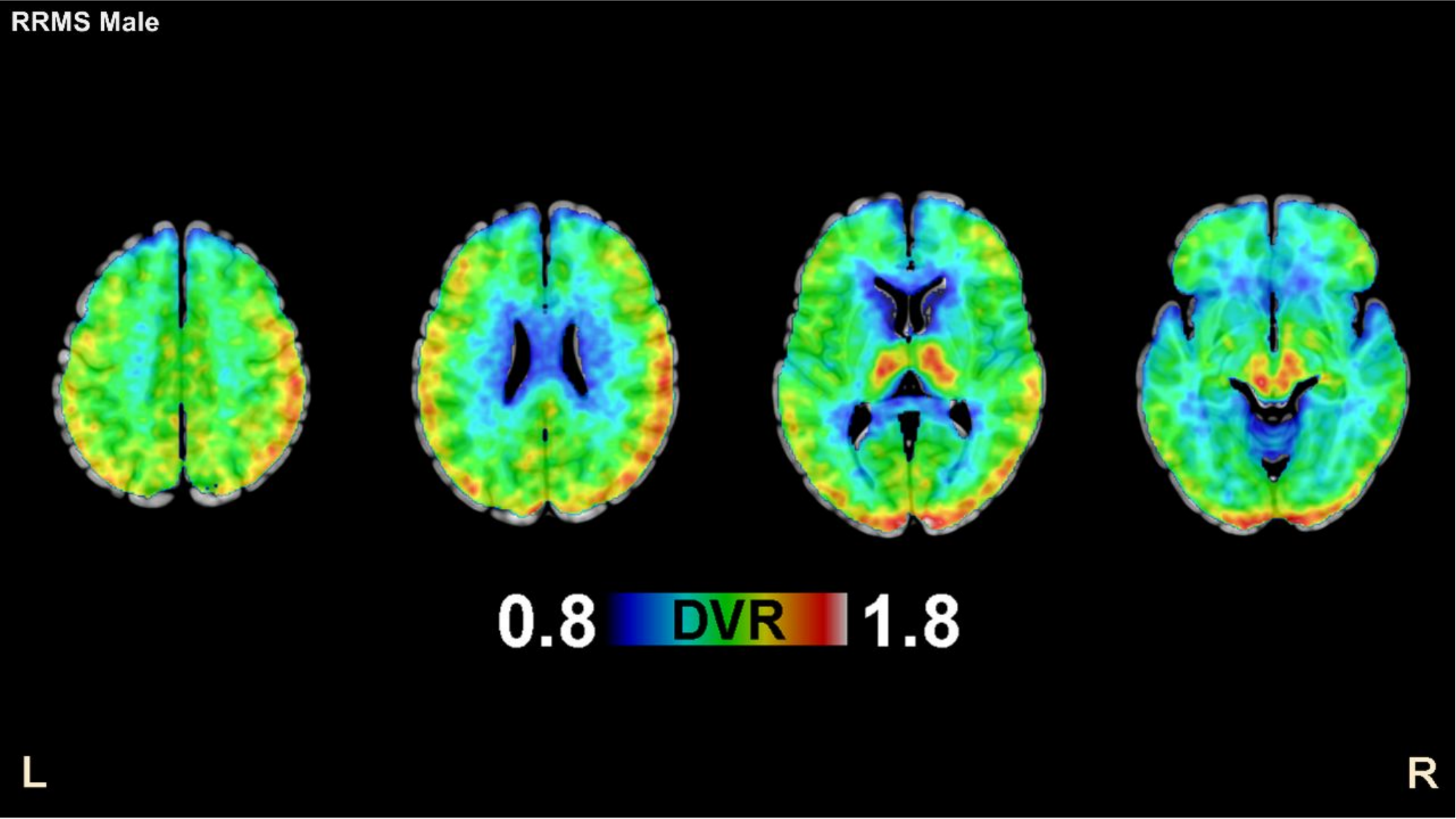

## Slide 6
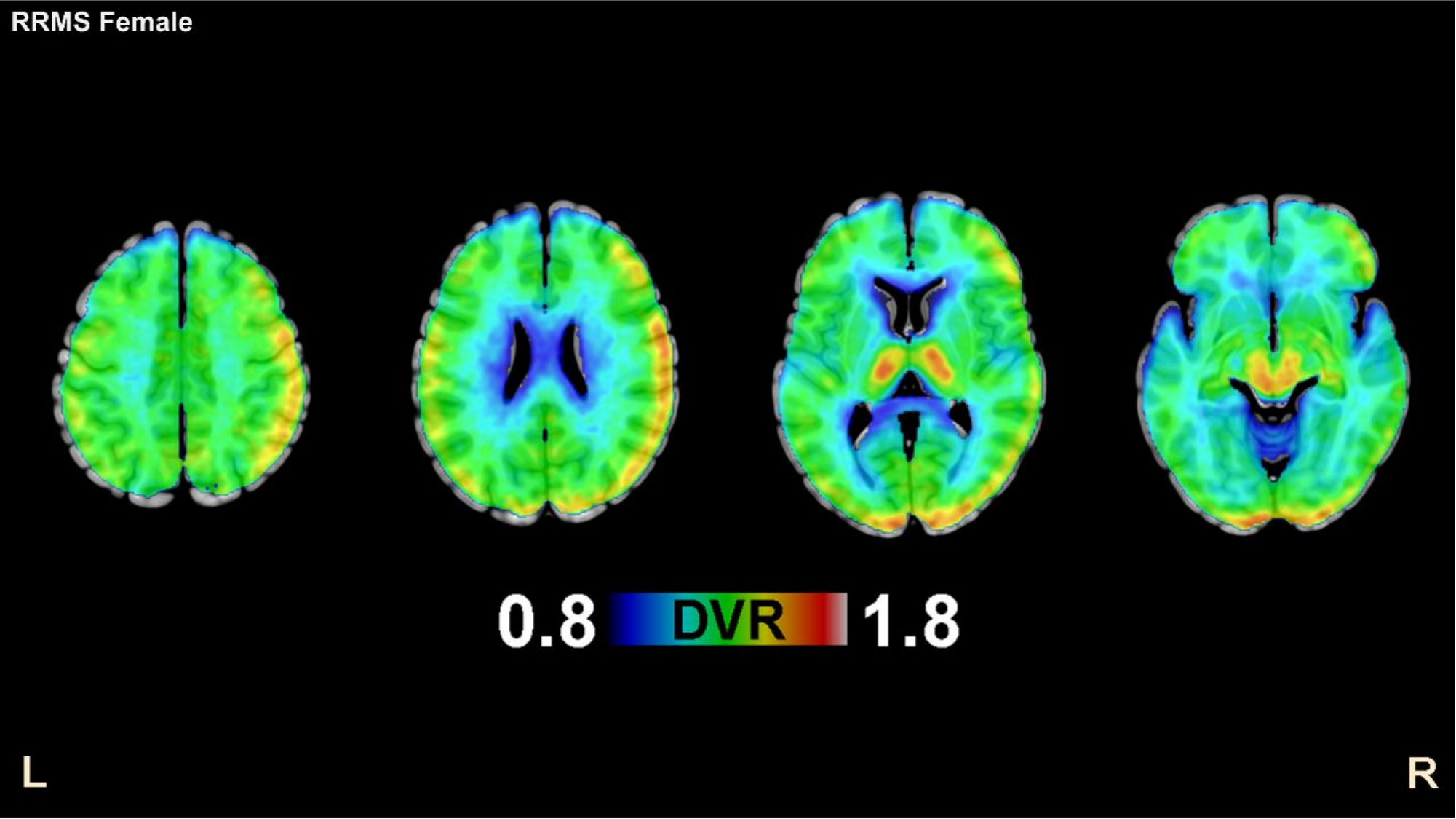

## Slide 7
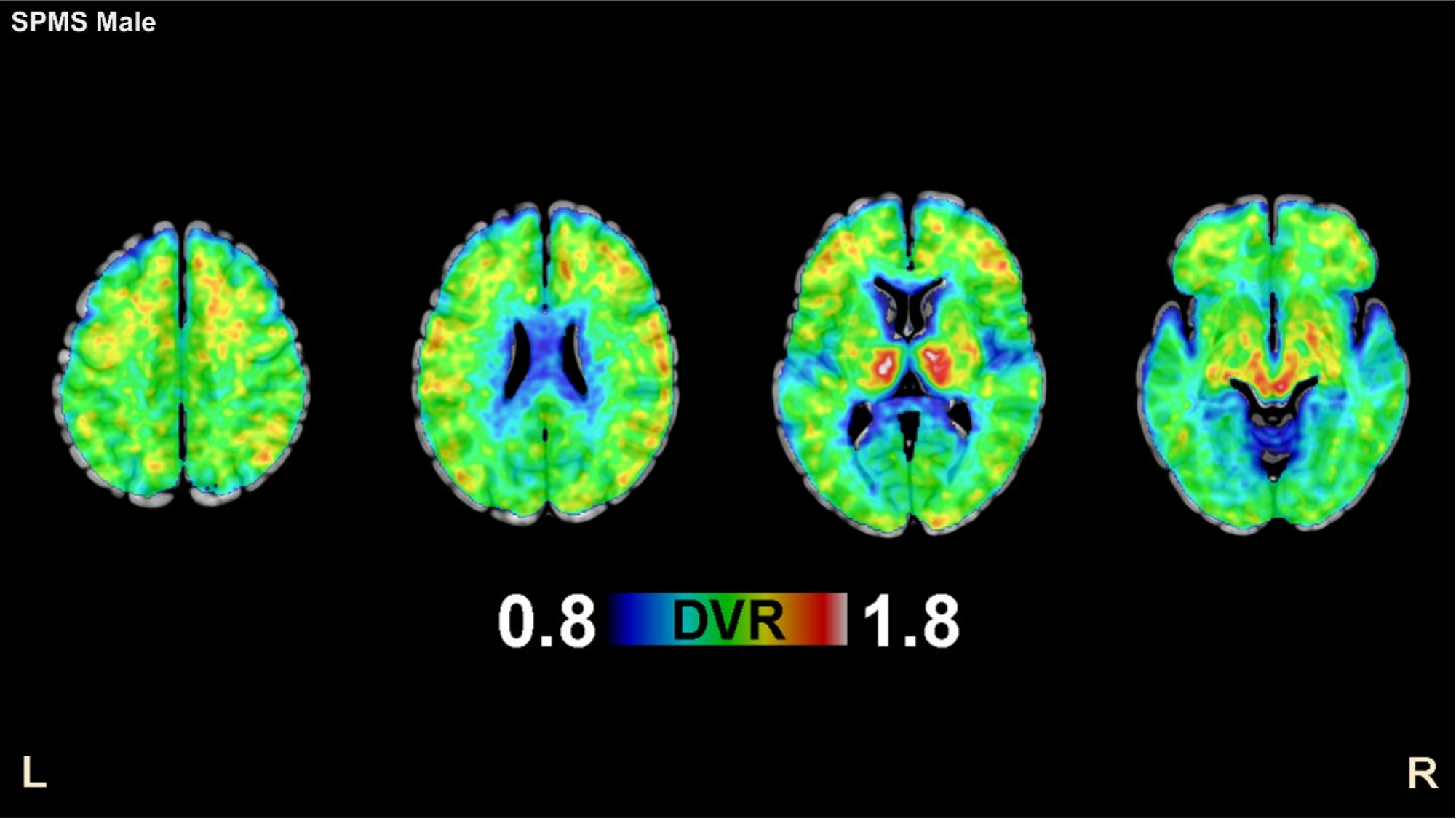

## Slide 8
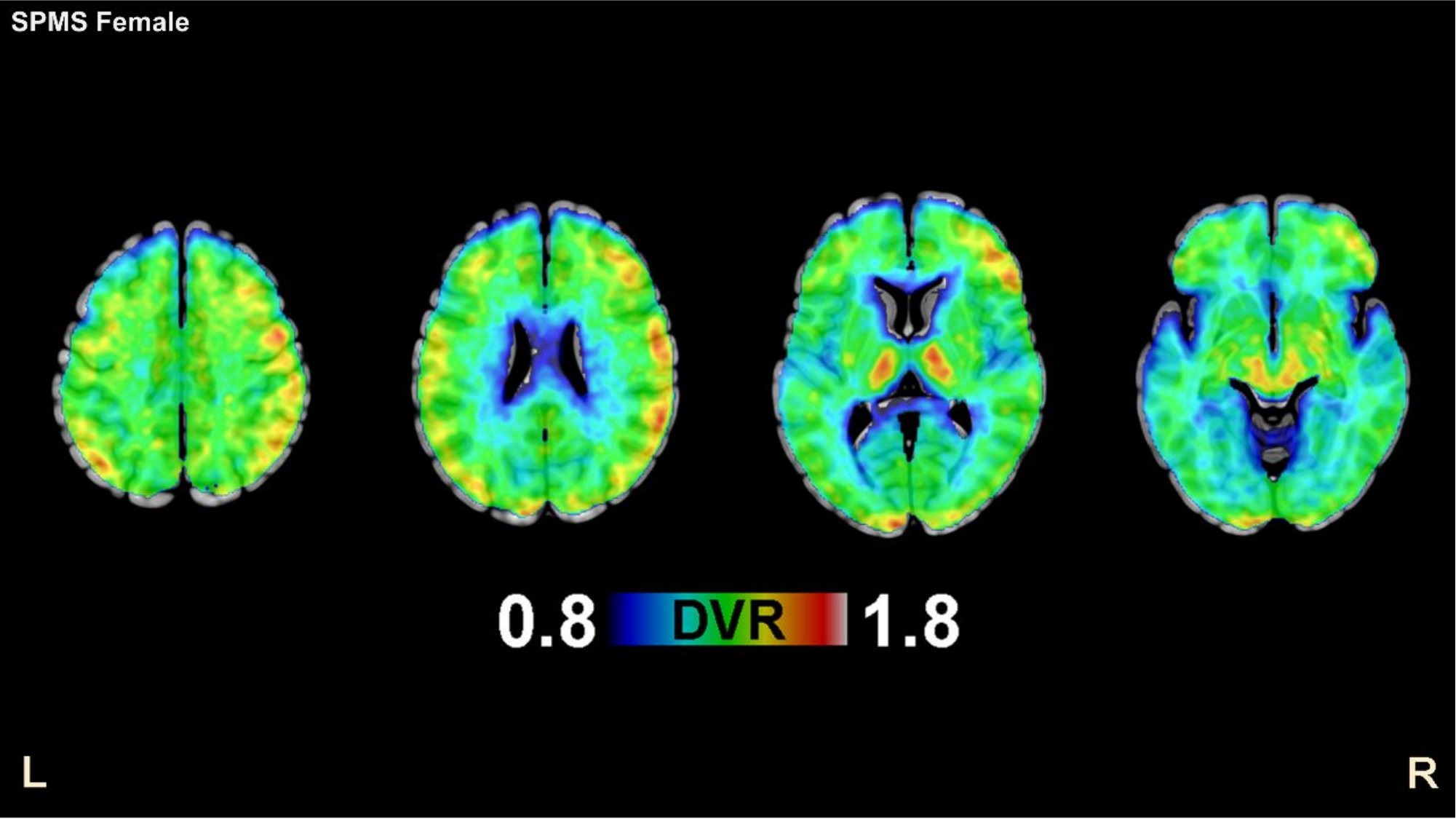

Supplement: Supplementary file 1 [file Presentation_1.PPTX]
